# Supplementary material for: Ethnic Disparities in Lipid Metabolism and Clinical Outcomes between Dutch South Asians and Dutch White Caucasians with Type 2 Diabetes Mellitus
Source: Metabolites. 2024 Jan 3;14(1):33. doi: 10.3390/metabo14010033 (PMC10819672; doi:10.3390/metabo14010033)
Supplement: Supplementary file 1 [file metabolites-14-00033-s001.zip › metabolites-2784019-supplementary/metabolites-2784019 - Table S1.pdf]

Table S1. Clinical characteristics of study participants.

	Dutch South Asian			Dutch white Caucasian			DSA-T2DM vs DwC-T2DM
	Control (n=21)	T2DM (n=47)	p-value[1]	Control (n=30)	T2DM (n=45)	p-value[1]	p-value[2]
**Demographics**							
Age (years)	48.3 (8.1)	54.9 (10.1)	0.0367	57.9 (7.9)	59.0 (6.5)	>0.9999	0.1606
Women (no, %)	15 (71.4%)	28 (59.6%)	0.3489	14 (46.7%)	20 (44.4%)	0.8498	0.1464
Current smoker (no, %)	3 (14.3%)	7 (14.9%)	1.0000	1 (3.3%)	9 (20.0%)	0.0437	0.7108
**Medical history diabetes**							
Duration diabetes mellitus (years)	-	17.9 (10)	-	-	10.3 (6.0)	-	<0.0001
Pan-microvascular (n, %)		30 (63.8%)	-		24 (51.1%)	-	0.3067
Nephropathy (n, %)	-	10 (21.3%)	-	-	11 (23.4%)	-	0.7174
Neuropathy (n, %)	-	14 (29.8%)	-	-	15 (31.9%)	-	0.7144
Retinopathy (n, %)	-	24 (51.1%)	-	-	5 (10.6%)	-	<0.0001
Macrovascular (n, %)	-	13 (27.7%)	-	-	2 (4.3%)	-	0.0037
**Medication use**			-			-	
Metformin (n, %)	-	45 (95.7%)	-	-	45 (100%)	-	0.4947
Sulfonylurea derivatives (n, %)	-	8 (17.0%)	-	-	13 (28.9%)	-	0.2682
Insulin (n, %)	-	36 (76.6%)	-	-	29 (64.4%)	-	0.2935
Anti-hypertensive medication (n, %)	-	34 (72.3%)	-	-	34 (75.6%)	-	0.7255
ACE-inhibitors (n, %)	-	13 (27.7%)	-	-	17 (37.8%)	-	0.4165
Statins (n, %)	-	36 (76.6%)	-	-	36 (80.0%)	-	0.8864
**Blood pressure**							
Systolic blood pressure (mmHg)	123.5 (13.7)	144.6 (21.5)	0.0003	126.2 (12.1)	141.3 (15.0)	0.0007	>0.9999
Diastolic blood pressure (mmHg)	80.2 (11.8)	85.3 (10.0)	0.1746	80 (77-83)	86.9 (8.8)	0.0158	0.8458
**Anthropometrics**							
Weight	63.6 (9.9)	79.7 (11.7)	<0.0001	73.3 (10.5)	96.4 (13.6)	<0.0001	<0.0001
BMI (kg/m^2)	23.5 (3.0)	29.5 (4.0)	<0.0001	24.3 (3.3)	32.3 (3.9)	<0.0001	0.0018
Total body fat (%)	32.4 (7.1)	37.1 (9.1)	0.1576	32.5 (7.1)	37.2 (9.3)	<0.0001	0.9998
Waist circumference, cm	82.0 (7.4)	101.0 (9.5)	<0.0001	86.6 (9.1)	110.4 (8.9)	<0.0001	<0.0001
Hip circumference, cm	95.2 (7.3)	104.1 (8.0)	<0.0001	98.2 (6.1)	107.6 (7.5)	<0.0001	0.1264
Waist-to-hip ratio	0.9 (0.1)	1 (0.1)	<0.0001	0.9 (0.1)	1 (0.1)	<0.0001	0.0039
VAT, cm^2	73.2 (29.8)	166.4 (55.8)	<0.0001	74.7 (34.1)	205.6 (75.6)	<0.0001	0.4342
SAT, cm^2	233.2 (195.9-258.8)	300.1 (228.3-371.4)	0.0561	189.7 (148.8-238.6)	335.7 (262.4-419.5)	<0.0001	>0.9999
VAT-SAT ratio	0.3 (0.2-0.4)	0.5 (0.4-0.7)	0.0017	0.4 (0.3-0.6)	0.6 (0.4-0.9)	0.0064	>0.9999
**Glycemic control**							
Fasting glucose (mmol/L)	5.0 (0.3)	8.1 (3.0)	<0.0001	5.2 (0.5)	7.8 (2.1)	<0.0001	>0.9999
HbA1c (mmol/mol)	35.5 (2.4)	67.8 (11.3)	<0.0001	35.5 (2.7)	64.9 (10.7)	<0.0001	>0.9999
**Lipid panels**							
Total cholesterol (mmol/L)	5.4 (0.8)	4.2 (0.9)	<0.0001	5.7 (1.1)	4.8 (1.0)	0.0018	0.0226
HDL-cholesterol (mmol/L)	1.6 (0.3)	1.2 (0.3)	0.0028	1.9 (0.5)	1.3 (0.3)	<0.0001	0.9008
LDL-cholesterol (mmol/L)	3.4 (0.7)	2.1 (0.8)	<0.0001	3.3 (1.0)	2.6 (0.8)	0.0017	0.0416
Total Triglycerides (mmol/L)	0.9 (0.3)	1.8 (1.4)	0.0031	0.9 (0.7-1.2)	2.1 (1.3)	<0.0001	0.2758
**Kidney function panels**							
Serum creatinine (μmol/mL)	68.0 (60.0-79.0)	67.0 (59.0-83.5)	0.4096	73.0 (68.0-85.0)	68.0 (57.0-80.0)	0.9582	0.9823
Albumin/creatine ratio (mg/mmol)	-	2.7 (0.55-8.45)	-	-	0.7 (0-2.5)	-	0.0037
Micro-albuminuria (n, %)[a]	-	15 (31.9%)	-	-	7 (15.6%)	-	-
Macro-albuminuria (n, %)[b]	-	7 (14.9%)	-	-	1 (2.2%)	-	-

Data are presented as mean (SD), median (25-75 percentile), or percentage.

Abbreviations: *ACE* Angiotensin-converting enzyme, *BMI* body mass index, *HbA1c* Hemoglobin A1c, *HDL* high-density lipoprotein, *LDL* low-density lipoprotein, *SAT* subcutaneous adipose tissue, *VAT* visceral adipose tissue.

[1]Post hoc tests of unpaired One-way ANOVA, Kruskal-Wallis test, or Chi-square test, $p<0.05$

[2]Post hoc tests of unpaired One-way ANOVA or Kruskal-Wallis test, Chi-square test, or Fisher's exact test, $p<0.05$

[a]Albumin-creatinine ratio between 3.0 – 30 mg/mmol. [b]Albumin-creatinine ratio > 30 mg/mmol

Most of these data have been published before [16, 17].

16. Bizino, M.B.; Jazet, I.M.; Westenberg, J.J.M.; van Eyk, H.J.; Paiman, E.H.M.; Smit, J.W.A.; Lamb, H.J. Effect of liraglutide on cardiac function in patients with type 2 diabetes mellitus: randomized placebo-controlled trial. *Cardiovasc Diabetol* **2019**, *18*, 55, doi:10.1186/s12933-019-0857-6.
17. van Eyk, H.J.; Paiman, E.H.M.; Bizino, M.B.; de Heer, P.; Geelhoed-Duijvestijn, P.H.; Kharagjitsingh, A.V.; Smit, J.W.A.; Lamb, H.J.; Rensen, P.C.N.; Jazet, I.M. A double-blind, placebo-controlled, randomised trial to assess the effect of liraglutide on ectopic fat accumulation in South Asian type 2 diabetes patients. *Cardiovasc Diabetol* **2019**, *18*, 87, doi:10.1186/s12933-019-0890-5.
