# Supplementary material for: Ethnic Disparities in Lipid Metabolism and Clinical Outcomes between Dutch South Asians and Dutch White Caucasians with Type 2 Diabetes Mellitus
Source: Metabolites. 2024 Jan 3;14(1):33. doi: 10.3390/metabo14010033 (PMC10819672; doi:10.3390/metabo14010033)
Supplement: Supplementary file 1 [file metabolites-14-00033-s001.zip › metabolites-2784019-supplementary/metabolites-2784019-supplementary.pdf]

Supplemental material

Ethnic Disparities in Lipid Metabolism and Clinical Outcomes between Dutch South Asians and Dutch White Caucasians with Type 2 Diabetes Mellitus

Lushun Yuan [1,2,†], Aswin Verhoeven [3], Niek Blomberg [3], Huub J. van Eyk [4], Maurice B. Bizino [5], Patrick C. N. Rensen [1,4], Ingrid M. Jazet [4], Hildo J. Lamb [5], Ton J. Rabelink [1,2], Martin Giera [3] and Bernard M. van den Berg [1,2,*]

[1] Einthoven Laboratory for Vascular and Regenerative Medicine, Leiden University Medical Center, 2333 ZA Leiden, The Netherlands

[2] Department of Internal Medicine, Division of Nephrology, Leiden University Medical Center, 2333 ZA Leiden, The Netherlands

[3] Center for Proteomics and Metabolomics, Leiden University Medical Center, 2333 ZA Leiden, The Netherlands

[4] Department of Internal Medicine, Division of Endocrinology, Leiden University Medical Center, 2333 ZA Leiden, The Netherlands

[5] Department of Radiology, Leiden University Medical Center, 2333 ZA Leiden, The Netherlands

* Correspondence: b.m.van_den_berg@lumc.nl

† Current address: Department of Vascular Surgery, Intervention Center, Shanghai General Hospital, Shanghai Jiao Tong University, Shanghai 201620, China.

Supplementary figures

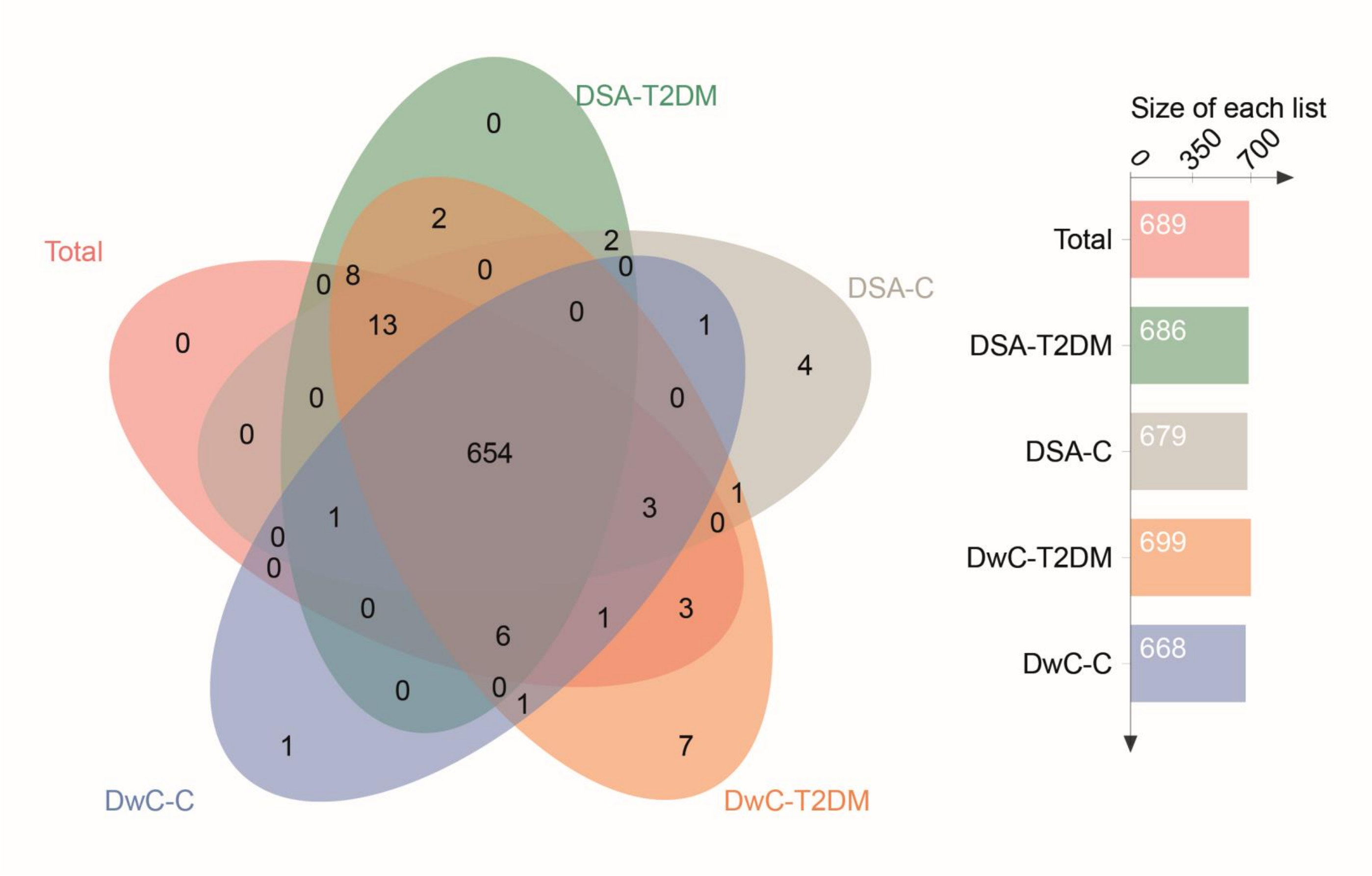

Supplementary Figure S1. Lipid species selected for analysis. Missing value percentage quantification per lipid class in total individuals, Dutch South Asians with T2DM (DSA-T2DM), Healthy Dutch South Asians (DSA-C), Dutch white Caucasians with T2DM (DwC-T2DM), and healthy Dutch white Caucasians (DwC-C). Details of missing values are shown in Additional file 2: Table S2. Common lipids with a missing value percentage of less than 30%.

Abbreviations: DSA Dutch South Asian; DwC Dutch white Caucasian; T2DM type 2 diabetes mellitus; TG triglyceride.

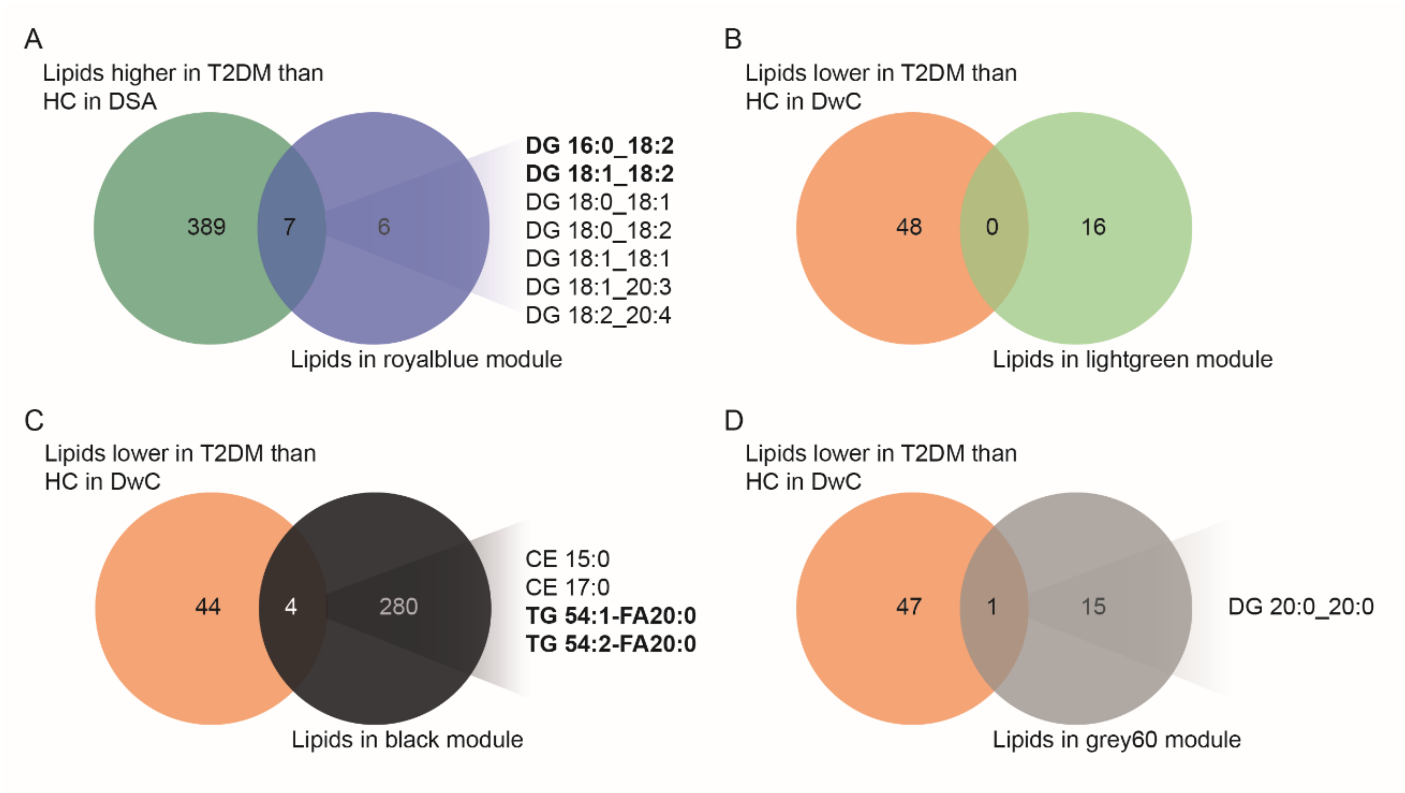

Supplementary Figure S2. Identification of key mediatory lipids. (A) Key mediatory lipid species (from diabetic nephropathy [DN] and glycemic control associated module, 'royal blue' module) in Dutch South Asians. (B) Key mediatory lipid species (from DR-associated module, 'light green' module) in Dutch white Caucasians. (C) Key mediatory lipid species (from diabetic retinopathy [DR]-associated module, 'black' module) in Dutch white Caucasians. (D) Key mediatory lipid species (diabetic nephropathy [DN]-associated module, 'grey60' module) in DwC. Bold lipids indicated that they were specifically different in DSA/DwC.

Abbreviations: CE cholesteryl ester; DG diacylglyceride; DN diabetic nephropathy; DR diabetic retinopathy; DSA Dutch South Asian; DwC Dutch white Caucasian; HC healthy control; T2DM type 2 diabetes mellitus; TG triglyceride.

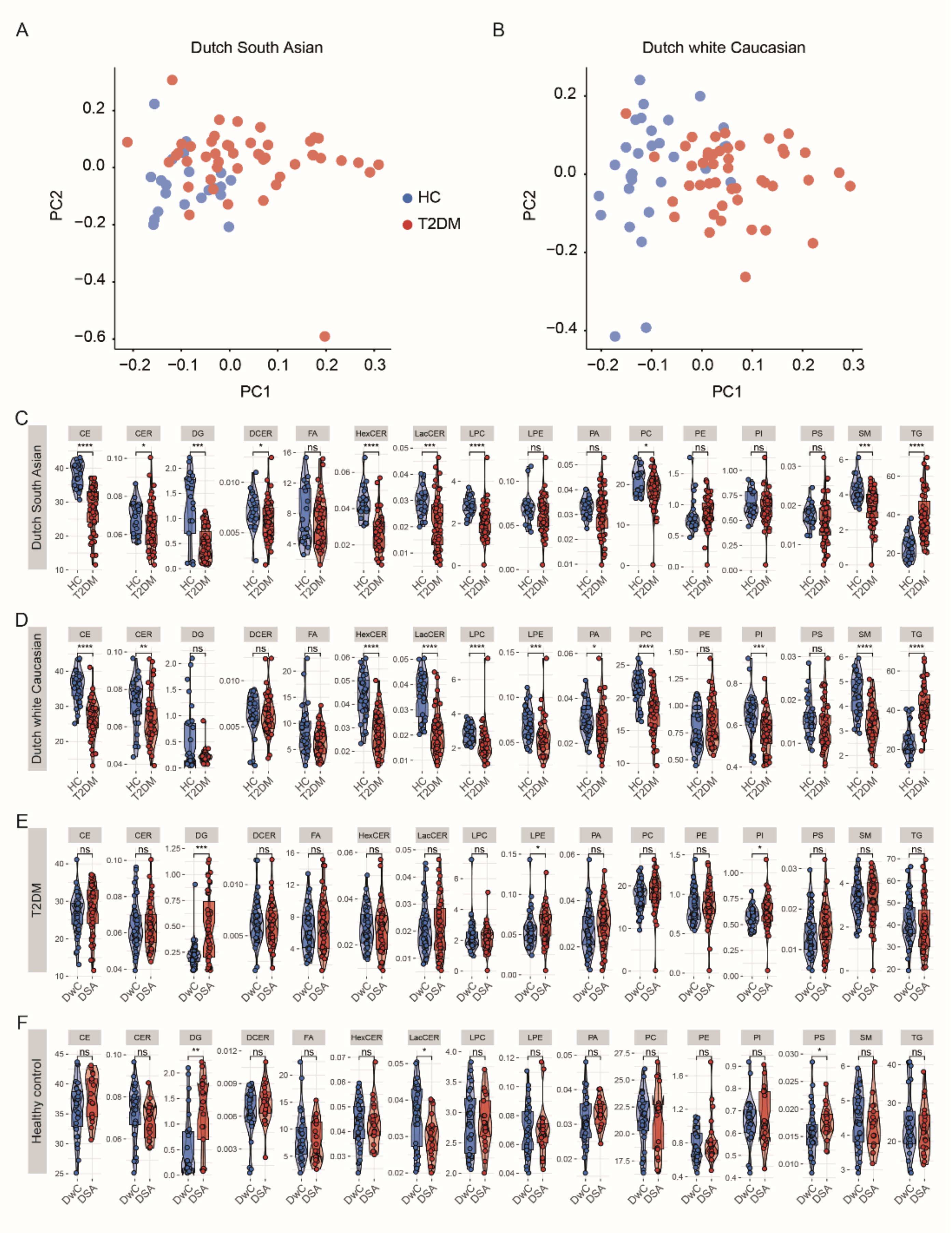

Supplementary Figure S3. Lipid abundance comparison between individuals from two ethnic groups. Principle component analysis using lipid class abundance in (A) Dutch South Asians and (B) Dutch white Caucasians. (C) Violin plot of lipid class abundance between patients with T2DM and healthy controls in Dutch South Asians. (D) Violin plot of lipid class abundance between patients with T2DM and healthy

controls in Dutch white Caucasians. (E) Violin plot of lipid class abundance between Dutch South Asians with T2DM and Dutch white Caucasians with T2DM. (F) Violin plot of lipid class abundance in healthy individuals between Dutch South Asians and Dutch white Caucasians. Wilcoxon signed-rank test was performed; *$p<0.05$, **$p<0.01$, ***$p<0.001$, ****$p<0.0001$.

Abbreviations: CE cholesteryl ester; CER ceramide; DCER dihydroceramide; DG diacylglyceride; DSA Dutch South Asian; DwC Dutch white Caucasian; FA fatty acid; HC healthy control; HexCER hydroxyceramide; LacCER lactosylceramide; LPC lysophosphatidylcholine; LPE lysophosphatidylethanolamine; PA phosphatidic acid; PC phosphatidylcholine; PE phosphatidylethanolamine; PI phosphatidylinositol; PS phosphatidylserine; SM sphingomyelin; T2DM type 2 diabetes mellitus; TG triglyceride.

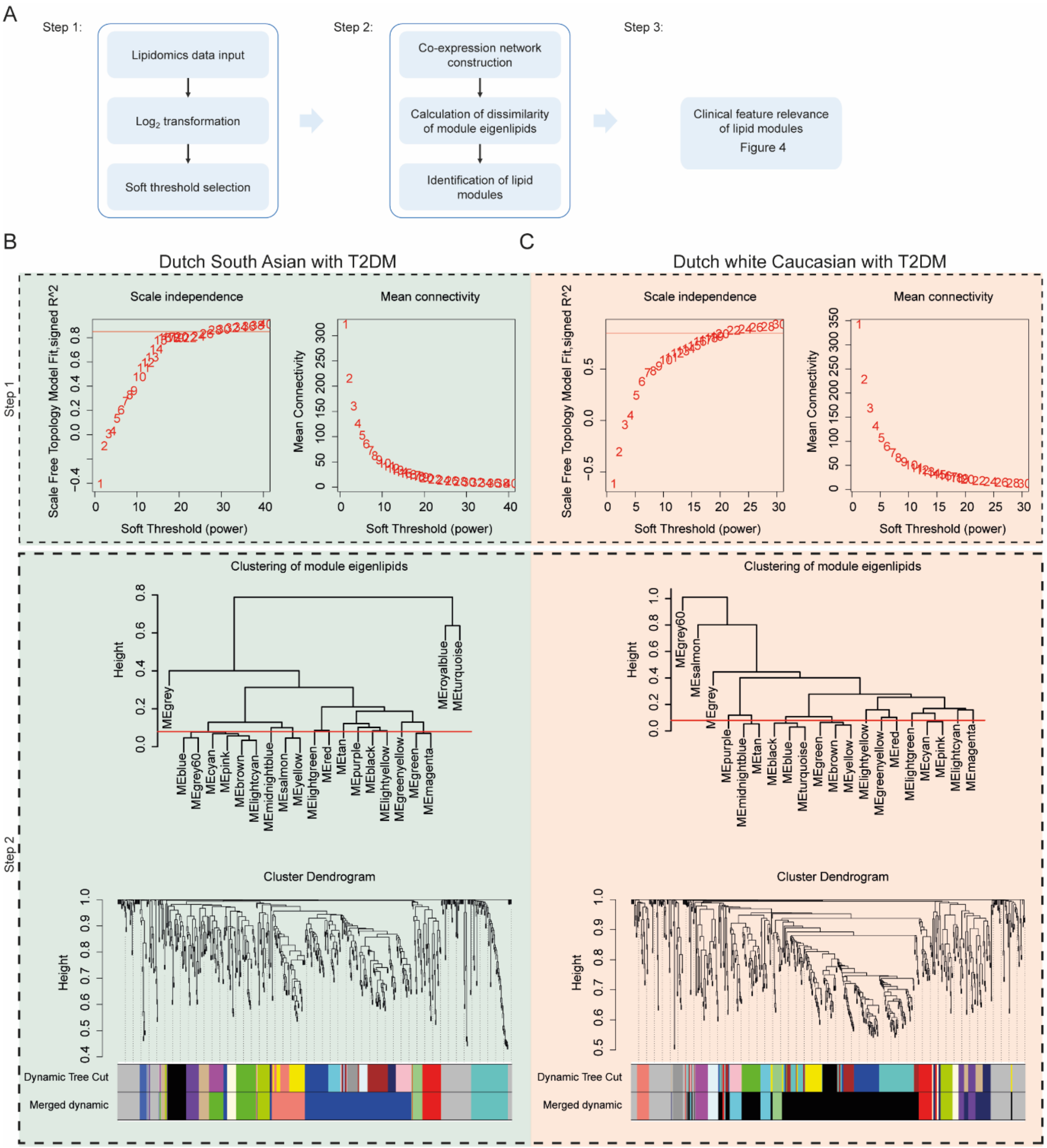

Supplementary Figure S4. Step-by-step WGCNA analysis. (A) Workflow of WGCNA analysis using the lipidomics profiles. Step 1 and Step 2 of WGCNA analysis in (B) Dutch South Asian and (C) Dutch white Caucasian.

Abbreviations: *ME* module eigenlipids

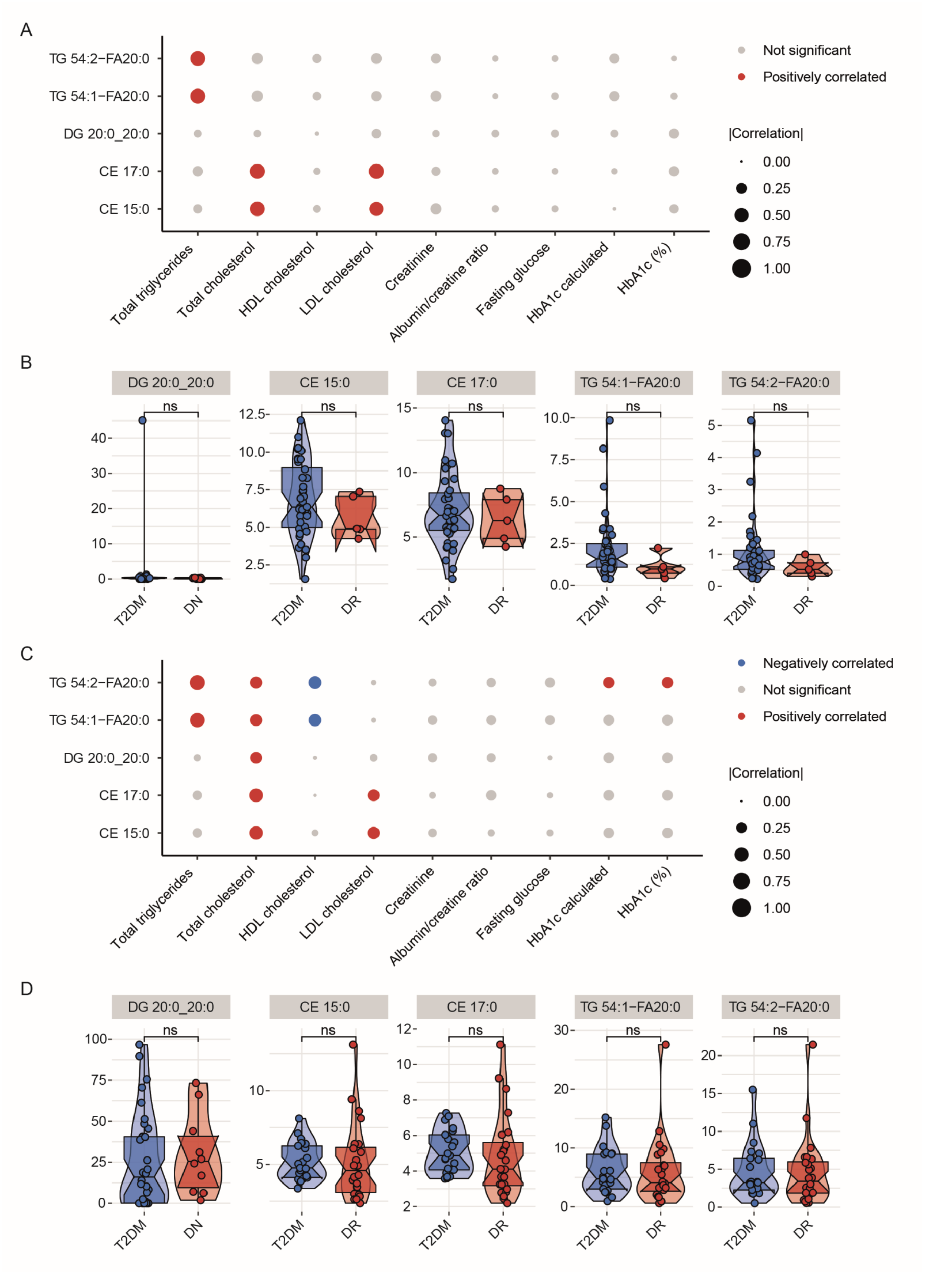

Supplementary Figure S5. Correlations between key mediatory lipids in Dutch white Caucasians and dyslipidemia, kidney function, and glycemic control. (A) A bubble plot depicting the correlations of lipids with lipoproteins, kidney function, and glycemic control in Dutch South Asians with T2DM. (B) Violin plot

of lipids between T2DM with and without DN/DR in Dutch South Asians. (C) A bubble plot depicting the correlations of lipids with lipoproteins, kidney function, and glycemic control in Dutch South Asians with T2DM. (D) Violin plot of lipids between T2DM with and without DN/DR in Dutch South Asians. The colour grey indicates no significant correlations with clinical features, the colour blue indicates a negative correlation with clinical features, and the colour red indicates a positive correlation with clinical features. The size of the dots represents the correlation coefficients (Pearson's correlation). The Wilcoxon signed-rank test was performed.

Abbreviations: CE cholesteryl ester; DG diacylglyceride; DN diabetic nephropathy; DR diabetic retinopathy; HbA1c hemoglobin A1c; HDL high-density lipoprotein; LDL low-density lipoprotein; ns not significant; T2DM type 2 diabetes mellitus; TG triglyceride.

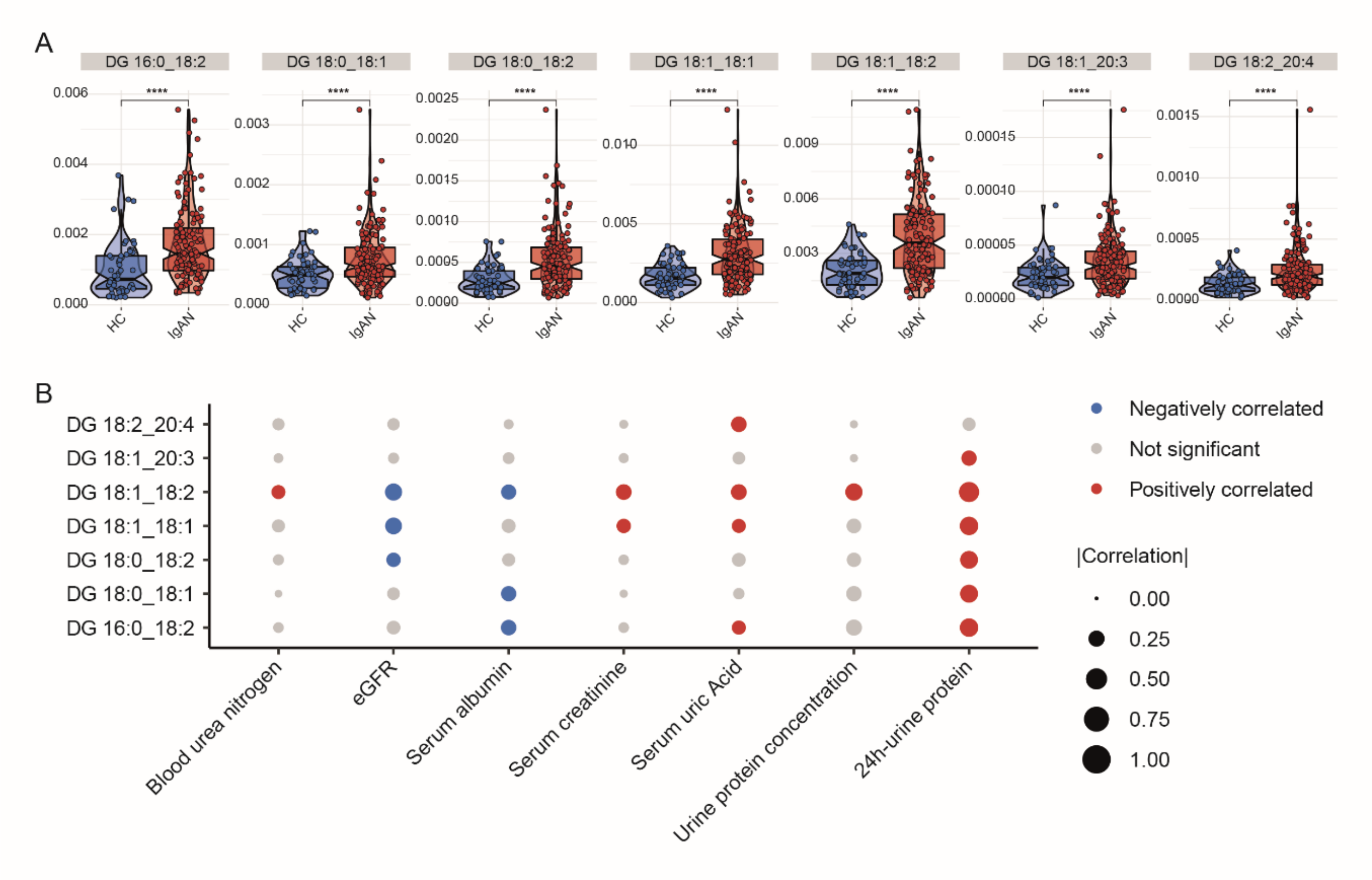

Supplementary Figure S6. The role of key mediatory lipids in DN-associated module of DSA in IgA nephropathy and their relationships with kidney function. (A) Violin plot of lipid differences between healthy controls and IgA nephropathy. (B) A bubble plot illustrating the relationships between the key mediatory lipids of Dutch South Asians and kidney function parameters in IgA nephropathy. The colour grey denotes no significant correlations with clinical features, the colour blue denotes a negative correlation with clinical features, and the colour red denotes a positive correlation with clinical features. The correlation coefficients (Pearson's correlation) are represented by the size of the dots. The Wilcoxon signed-rank test was performed; ****p<0.0001.

Abbreviations: CE cholesteryl ester; DG diacylglyceride; eGFR estimated glomerular filtration rate; HC healthy control; IgAN IgA nephropathy.

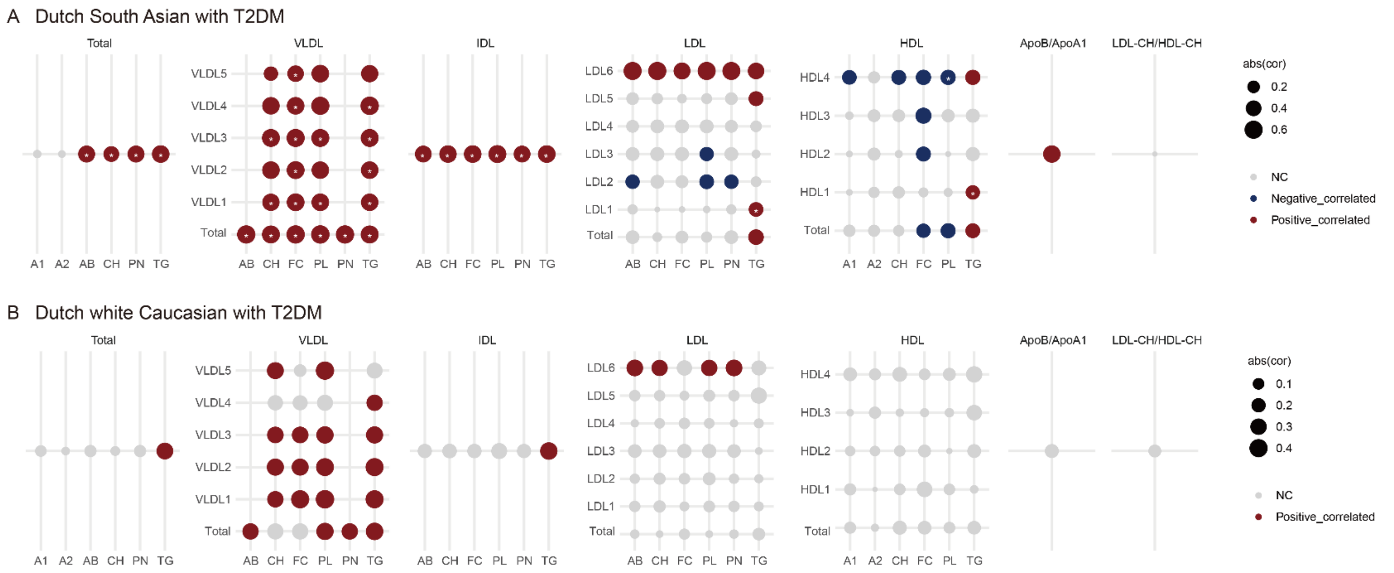

Supplementary Figure S7. Correlation of DG 18:1_18:2 with ^{1}H NMR- generated lipoproteins and lipoprotein subfractions**.** (A) A bubble plot illustrating the relationships between DG 18:1_18:2 and lipoproteins and lipoprotein subfractions in Dutch South Asian with T2DM. (B) A bubble plot illustrating the relationships between DG 18:1_18:2 and lipoproteins and lipoprotein subfractions in Dutch white Caucasian with T2DM. The colour grey denotes no significant correlations, blue colour denotes a negative correlation, and red colour denotes a positive correlation. The correlation coefficients (Pearson's correlation) are represented by the size of the dots. The Wilcoxon signed-rank test was performed; *p<0.05.

Abbreviations: A1/ApoA1 apolipoprotein A1; A2 apolipoprotein A2; AB/ApoB apolipoprotein B; CH cholesterol; FC free cholesterol; PL phospholipid; PN particle number; TG triglyceride.
